# Estimating Determinants of Attrition in Eating Disorder Communities on Twitter: An Instrumental Variables Approach

**DOI:** 10.2196/10942

**Published:** 2019-05-03

**Authors:** Tao Wang, Emmanouil Mentzakis, Markus Brede, Antonella Ianni

**Affiliations:** 1 Department of Economics University of Southampton Southampton United Kingdom; 2 The Alan Turing Institute London United Kingdom; 3 Department of Electronics and Computer Science University of Southampton Southampton United Kingdom; 4 Department of Economics Università Cà Foscari Venice Italy

**Keywords:** medical informatics, eating disorders, social media, attrition, emotions, social network, causality, instrumental variables

## Abstract

**Background:**

The use of social media as a key health information source has increased steadily among people affected by eating disorders (EDs). Research has examined characteristics of individuals engaging in online communities, whereas little is known about discontinuation of engagement and the phenomenon of participants dropping out of these communities.

**Objective:**

This study aimed to investigate the characteristics of dropout behaviors among eating disordered individuals on Twitter and to estimate the causal effects of personal emotions and social networks on dropout behaviors.

**Methods:**

Using a snowball sampling method, we collected a set of individuals who self-identified with EDs in their Twitter profile descriptions, as well as their tweets and social networks, leading to 241,243,043 tweets from 208,063 users. Individuals’ emotions are measured from their language use in tweets using an automatic sentiment analysis tool, and network centralities are measured from users’ following networks. Dropout statuses of users are observed in a follow-up period 1.5 years later (from February 11, 2016 to August 17, 2017). Linear and survival regression instrumental variables models are used to estimate the effects of emotions and network centrality on dropout behaviors. The average levels of attributes among an individual’s followees (ie, people who are followed by the individual) are used as instruments for the individual’s attributes.

**Results:**

Eating disordered users have relatively short periods of activity on Twitter with one half of our sample dropping out at 6 months after account creation. Active users show more negative emotions and higher network centralities than dropped-out users. Active users tend to connect to other active users, whereas dropped-out users tend to cluster together. Estimation results suggest that users’ emotions and network centralities have causal effects on their dropout behaviors on Twitter. More specifically, users with positive emotions are more likely to drop out and have shorter lasting periods of activity online than users with negative emotions, whereas central users in a social network have longer lasting participation than peripheral users. Findings on users’ tweeting interests further show that users who attempt to recover from EDs are more likely to drop out than those who promote EDs as a lifestyle choice.

**Conclusions:**

Presence in online communities is strongly determined by the individual’s emotions and social networks, suggesting that studies analyzing and trying to draw condition and population characteristics through online health communities are likely to be biased. Future research needs to examine in more detail the links between individual characteristics and participation patterns if better understanding of the entire population is to be achieved. At the same time, such attrition dynamics need to be acknowledged and controlled when designing online interventions so as to accurately capture their intended populations.

## Introduction

### Background

Eating disorders (EDs), such as anorexia and bulimia, are complex mental disorders defined by extreme obsessions with body weight or shape and unusual eating behaviors [1]. These diseases have the highest mortality rate of any mental illness [2], intractable comorbidities, [3] and worldwide prevalence [4], having become a major public health concern. Although a variety of treatment options have emerged over recent years [5], populations affected by EDs are often hard to reach through traditional health care services. This is mainly because of fear of stigma or a feeling of shame; many sufferers conceal their ED symptoms and never seek professional treatment or support [6,7]. To keep struggles with illnesses private, people often seek health-related information and support through online peer-to-peer communities, particularly via social media sites such as Twitter and Facebook. Participation in online communities is common in ED populations [8] and has been suggested as a screening factor for EDs [3]. This provides an opportunity for health care professionals to deliver health support to these hard-to-reach populations through online communities [9-13]. Moreover, as online communities present a relatively anonymous environment for individuals to naturally self-disclose and socialize [14], user-generated data online provide a large amount of records about individuals’ concerns, thoughts, emotions, and social interactions [15-17], which can complement traditional data sources (eg, surveys and interviews) in understanding risk factors of EDs. Hence, growing research has focused on characterizing individuals’ behavioral patterns in online communities [15-19] so as to better understand EDs and promote population-level well-being.

One notable characteristic of online ED communities is their participants have widely different stances on EDs [8,20,21]. Some communities encourage members to discuss their struggles with EDs, share treatment options, and offer support toward recovery from EDs, so called *prorecovery* communities [20-22]. There are also many *anti-recovery* or *pro-ED* communities in which members often deny an ED to be a disorder and instead promote EDs as a healthy lifestyle choice [8,23]. These pro-ED communities can negatively affect health and quality of life among people with and without EDs, through reinforcing an individual’s identity around EDs [24], promoting thin ideals [25], and disseminating harmful practices for weight loss [8]. Recent studies have shown that individuals’ language use online strongly indicates their pro-ED or prorecovery stances [15,17,20], as well as emotions of depression, helplessness, and anxiety that reflect their mental disorders [16]. Other studies have also examined interactions between pro-ED and prorecovery communities on Flickr [21], anorexia-related misinformation [18], sentiments in comments on ED-related videos on YouTube [26], characteristics of removed pro-ED content, [27] and lexical variation of pro-ED tags on Instagram [19,28]. Yet, prior studies have largely focused on examining how people engage in and maintain an online ED community, whereas little is known about how people drop out of such a community. As a dynamic process, people who join and actively engage in a community at earlier stages can have less participation and leave the community at later stages. Understanding the attrition processes of online communities can enhance our knowledge of the dynamics in these communities.

Studying the attrition process of an online community can also have practical implications for disease prevention and health interventions. Given the ease of accessibility of social media for many individuals (eg, via mobile devices), increasing attention has focused on using online communities to deliver health interventions [9-13,29,30]. One of the most popular approaches is to deliver health lessons and behavior-changing instructions via online communities [9-13,29]. Although pilot studies based on small samples have demonstrated the effectiveness of these approaches in reducing body dissatisfaction and disordered eating [12,13], evidence from interventions for a variety of health behaviors (eg, smoking, diet, exercise, and sexual health) suggests that attrition (ie, participant loss) is one of the most common challenges in online interventions [10,29]. This is known as the *law of attrition* of online interventions [31]. A recent study has shown a high attrition rate in an online intervention for EDs [32], though this intervention is delivered via a purposely designed website rather than a general social media site. Thus, an important goal in conducting successful interventions via online communities is to improve members’ retention, as members who remain longer are more likely to receive these interventions and have more opportunities to promote a target behavior change. To achieve this goal, a critical first step is to understand what factors influence members’ retention in an online community.

Previous studies have shown that people’s decisions of retention or dropout in online communities are associated with a variety of factors [33,34], including personality traits (eg, shyness and the Big Five traits) [35,36], interests [37], recognition in a community [38-41], and support from others [42,43]. However, such an association is not adequate to conclude the presence of a causal relationship [44,45] between an individual’s attributes and her or his online participation. This is because an association can arise from non-causal relationships. For example, most previous studies focus on the use of self-reported surveys and rely on participants’ reports of their own personality, concerns, and behaviors [35,39,46]. This can introduce considerable retrospective bias and measurement errors, leading to a coincidental association between 2 unrelated variables, particularly in small samples. Even if variables are measured rather than self-reported [43,47], participation in an online community is inherently self-selected (eg, sharing common interests) and members can drop out for many different reasons (eg, effect of an online or offline event). Thus, unobservable factors (ie, confounding variables) may affect both a main predictor and participation outcomes, causing a spurious association. Moreover, in some cases reverse causality can lead to an association. For example, previous studies suggest that feelings of social isolation are linked to frequent social media use [35,48] whereas recent studies indicate that social media use is linked to increased feelings of social isolation [49]. Technically speaking, the issues of measurement errors, confounding variables, and reverse causality can cause endogeneity, which refers to an explanatory variable of interest being correlated with the error term in a regression model [44]. In these cases, traditional methods such as ordinary least squares (OLS) give biased and inconsistent estimates of the effect of interest. It is therefore not surprising that mixed results exist in previous studies. For example, a positive association between individuals’ expertise and online participation was found in a study by Tausczik and Pennebaker [46] whereas a negative association was found in another study by Cook et al [38].

### Objectives

This study aimed to estimate determinants of dropout in an online ED community, while addressing the endogeneity issues by using an instrumental variable (IV) approach [44]. Specifically, we analyzed tweeting activities for a large set of individuals who self-identified with EDs on Twitter for over 1.5 years and identified the presence of dropout if a user ceased to post tweets in the observation period. We explored determinants of a user’s dropout based on the incentive theory [34,50], which argues that people’s engagement in an activity can be driven by (a) intrinsic motivation which refers to doing something because it is interesting or enjoyable and (b) extrinsic motivation which refers to doing something because it earns an external reward. We focused on intrinsic motivation captured by personal emotions and extrinsic motivation captured by sociometric status in an online peer-to-peer community. Rather than using self-reports [35,39,46], we measured users’ emotions based on their emotional expressions in tweets using sentiment analysis techniques [51] and quantified users’ sociometric statuses by network centrality [52] in the social network of an ED community on Twitter. On the basis of these measured variables, IV estimators both for the decision to drop out and for the time to drop out were implemented to achieve consistent estimates of the effects of personal emotions and network centrality on dropout in an online ED community. To better understand the estimation results, we further examined heterogeneity in tweeting interests (ie, topics discussed in tweets) of users with differing levels of characteristics (eg, emotions) and dropout outcomes. To our knowledge, this study is the first to systematically characterize the determinants of dropout behaviors in online ED communities. A total of 3 research questions were examined: (a) what are the general characteristics of the attrition process in an online ED community? (b) how do intrinsic and extrinsic factors affect the decision of an individual to drop out of the community? and (c) how do these factors affect the duration of time until the occurrence of dropout?

## Methods

### Data Collection

Our data are collected from Twitter, a microblogging platform that allows millions of users to self-disclose and socialize. As many social media platforms such as Facebook and Instagram have taken moderation actions to counteract pro-ED content and user accounts [28], Twitter has not yet enforced actions to limit such content [53]. This makes Twitter a unique platform to study the attrition process naturally happening in an online ED community and allows us to examine individuals’ behaviors in a nonreactive way. Our study protocol was approved by the Ethics Committee at the University of Southampton. All data used in our study are *public* information on Twitter and available through the Twitter application programming interfaces (APIs). No personally identifiable information was used in this study. Our data collection process included 3 phases:

1. First, we collected a set of individuals who self-identified with EDs on Twitter using a snowball sampling approach. Specifically, we tracked the public tweet stream using “eating disorder,” “anorexia,” “bulimia,” and “EDNOS” (ie, eating disorder not otherwise specified) from January 8 to 15, 2016. This resulted in 1169 tweets that mentioned EDs. From the authors of these tweets, we identified 33 users who self-reported both ED-related keywords (eg, “eating disorder,” “anorexia,” and “bulimia”) and personal bio-information (eg, body weight and height) in their profile descriptions (ie, a sequence of user-generated text describing their accounts below profile images). Starting from these seed users, we expanded the user set using snowball sampling through their social networks of followees and followers. At each sampling stage, we filtered out non-English speaking accounts and finally obtained 3380 unique ED users who self-reported ED-related keywords and bio-information in their profile descriptions. Note that our focus in this work is studying individuals who are affected by EDs rather than those who are related to EDs. The inclusion of bio-information in user sampling allowed us to filter out ED-related therapists, institutes, or organizations, as these users often displayed ED-related keywords but did not show bio-information in their Twitter profile descriptions. Details about the data collection of ED users can be found in our previous study [54].
2. Then, we collected all friends (including followees and followers) of each ED user, leading to a large social network consisting of 208,063 users. For each user, we retrieved up to 3200 (the limit returned from Twitter APIs) of their most recent tweets and obtained 241,243,043 tweets in total. The data collection process finished on February 11, 2016.
3. Finally, we opened a follow-up observation period for all users on August 17, 2017, to obtain measurements on users’ activities online. In the second observation, we only collected users’ profile information which includes users’ last posted statuses.

To verify the quality of our collected sample, 2 members of the research team classified a random sample of 1000 users on whether they were likely to be a true ED user based on their posted tweets, images, and friends’ profiles. Users were classified as “disordered” if they frequently and intensively posted their body weights, details of their dietary regimen (eg, calories), struggles with eating (eg, “I want to eat but cannot”), pictures of themselves, and self-reports of being disordered or in recovery in tweets and followed ED-related friends (eg, user profiles with ED-related keywords). The process revealed a 95.2% match between the identified ED individuals in the data collection stage and those classified as ED during inspection. Although it is impossible to diagnose individuals’ disorders based on their online behaviors, this inspection provides a strong indication that the collected users are likely to be affected by EDs rather than those who merely talk about EDs online. See [54] for details of data validation.

### Estimation Framework

There are 2 different models specified to estimate the effects of emotions and network centrality on dropout. First, we specified a linear probability model on the whole sample to estimate the effects of individuals’ characteristics observed in the first observation period on the probability of dropping out in the second observation period. Finally, we estimated survival models to explore the effects of individuals’ characteristics observed in the first observation on the time to dropout in the second observation (ie, the duration from our first observation to the dropout in our second observation). However, similar to all social media studies, only a limited number of individuals’ characteristics are available for our estimations and these are mostly observed through user-generated data online. This leads to confounding variable bias, as unobservable factors can be correlated with both the main explanatory variables (ie, emotions and network centrality) and dropout outcomes. For example, undergoing hospital treatment can simultaneously affect a person’s emotional state and the use of social media. Furthermore, previous studies have shown that social media use is associated with increased depression [55], social anxiety, [49] and body dissatisfaction [56,57], implying an effect of online participation on individuals’ emotions (ie, reverse causality). Both confounding variables and reverse causality result in biased estimates of the effects of emotions and network centrality on dropout. This problem can be addressed by using a randomized controlled trial in which emotions or network centralities are randomly assigned to users by researchers [58]. Such a trial, however, is not feasible, because of ethical and practical limitations [59].

Here, we utilized an alternative approach for estimating the effects of interest that is based on IV regression, an econometric technique to infer causal relations from observational data [44]. This technique has been applied to a variety of contexts, from identifying the causal effect of education on earning [60], the effect of a health treatment [61], to estimating social contagion effects on both online [59] and offline behaviors [62]. Formally, consider a model Y=Beta1*X1 + Beta2*X2 + u, where *X1* is endogenous, *X2* is exogenous, *u* is a random error term, and *Beta*s are effects to be estimated. IV methodology uses an instrument *Z* (which is [i] not contained in the explanatory equation, [ii] correlated with *X1*, namely *cov(Z,X1)* is not equal to 0, and [iii] uncorrelated with *u*, ie, *cov(Z,u)=0*, conditional on the other covariates such as *X2*) and runs a first stage reduced-form regression X1 = B1*Z + B2*X2 + v, where *v* is a random error and *B* s are coefficients. The causal effect of *X1* on *Y* is then given in a second stage regression Y=Beta1*X3 + Beta2*X2 + u, where *X3* is the predicted value of *X1* from the first stage. See [44] for more details.

### Measures

A number of variables are needed for estimations. All independent variables and IVs are measured in the first observation period (unless otherwise stated), whereas dependent variables are measured in the second observation period.

### Dropout Outcomes as Dependent Variables

Following previous studies [42,43], we identified the presence of dropout if a user ceased to post tweets. Specifically, in the linear probability models, we encoded the dropout status of a user as 0 (denoting *nondropout*) if the user had updated posts in our second observation and 1 (denoting *dropout*) otherwise.

In the survival models, each user has a 2-variable outcome: (a) a censoring variable denoting whether the event of dropout occurs and (b) a variable of survival time denoting the duration of time until the occurrence of dropout. We censored the occurrence of a “dropout event” in 2 ways. First, users are said to drop out if they have not posted tweets for more than a fixed threshold interval *π* before our second observation (so called *identical interval censoring*). As people use social media platforms with different activity levels (eg, some users post every several hours whereas other users only post once every couple of days), our second censoring method further accounts for personalized posting activities of individuals (called *personalized interval censoring*). In this method, users are said to drop out if they have not posted tweets for more than a variate threshold interval *λπ* + (1–*λ*) *Ii* before our second observation, where *π* is a fixed threshold, *Ii* is the average posting interval of individual *i* in our first observation period, and *λ* is a tunable parameter to control the effects of individual activities. We tuned the parameters by maximizing the agreement between the estimated dropout states based on users’ activities in our first observation and the observed states in our second observation. See Multimedia Appendix 1 for details. For users who were censored as dropped-out, we set their survival times as the durations from our first observation to their last postings in our second observation. For those who were censored as non-dropped-out, we set their survival times as the whole time period between our 2 observations.

### Emotions and Network Centrality as Main Explanatory Variables

Individuals’ emotions were measured through their language used in tweets. There is a variety of sentiment analysis algorithms to measure emotional expressions in texts [51,63]. In this study, we used SentiStrength [51] as (a) it has been used to measure the emotional content in online ED communities and has shown good interrater reliability [26] and (b) it is designed for short informal texts with abbreviations and slang and is thus suitable to process tweets [51]. After removing mention marks, hashtags, and URLs, each tweet was assigned a scaled value ranging from −4 to 4 by SentiStrength, where negative and positive scores indicate the strength of negative and positive emotions, respectively, and 0 denotes neutral emotions. We quantified a user’s emotional state by the average score of all tweets posted by the user. All retweets were excluded, as retweets reflect the emotions of their original authors more than those of their retweeters. To obtain robust results from the language processing algorithms, we only considered users who had more than 10 tweets and posted more than 50 words.

Network centrality measures the importance of a person in a social network; people well-recognized by their peers often have high centralities in a group [52]. To measure a user’s centrality in the ED community, we built a who-follows-whom network among ED users and their friends, where a directed edge runs from node A representing user A to node B representing user B if A follows B on Twitter. Although there are various measures of network centrality, we focused on coreness centrality [64] as it has been shown to outperform other measures such as degree and betweenness centrality [52] in detecting influential nodes in complex networks [65] and cascades of users leaving an online community [66,67]. We measured the sociometric status of a user in the ED community by the in-coreness centrality [68] of a node in the generated network using the package igraph version 0.7.0 [69].

### Aggregated Emotions and Network Centrality of Friends as Instrumental Variables

As IVs for a user’s attributes, we used average emotions and network centrality over all followees of the user, namely people who are followed by the user. The choice of these IVs is based on the following considerations. First, we considered the relevance assumption of our instruments, requiring that the characteristics of followees be correlated to the user’s characteristics, namely *cov (Z,X1)* is not equal to 0. We expected the followees’ updates to act as information sources for a user and followees’ behaviors as well as emotions manifested in their tweets can influence the user. Previous work [54] has shown the presence of homophily among ED users on Twitter suggesting that users who share similar emotional and network attributes tend to follow one another. Furthermore, the empirical existence and strength of the relevance property are tested in a first-stage regression and presented along with the structural estimates of the models.

Finally, we examined the exogeneity requirement (ie, *cov(Z,u)=0*), where followees’ emotions and centrality must not have a direct effect on the dropout decision of the user other than through their effect on the user’s emotions. Although we have taken such assumptions to be reasonable, we identified a pathway through which direct links could arise. Followees’ attributes (eg, emotions) could affect a user’s dropout through their effects on followees’ own dropouts. For example, followees’ emotional states may affect their own dropouts, and a feeling of loneliness because of friends’ leaving may then drive the target user to drop out. To control for this channel, we measured the proportion and durations of followees that remained active in our second observation (regardless of whether the target user dropped out or not). Furthermore, we changed the definition of followees (that are used to create the instruments) to those who are followed by a user but do not follow the user back (called *single way followees*). In this setting, the reverse causality of a user’s dropout on followees’ attributes was nullified, which strengthened the exogeneity assumption on IVs and controls.

### Estimation Covariates

Our estimates control for several covariates that may affect users’ tweeting activities, as listed in Table 1. First, we measured users’ social capital on Twitter (eg, the numbers of social connections and the levels of engagement in sharing content) to capture the fact that people with different levels of popularity may have different tendencies to share content online [70]. Note that, although the numbers of followees and followers can be regarded as the in- and out-degree centralities of a user in the whole social network on Twitter (ie, the “global” social capital), we were interested in the “local” network centrality in the ED-specific communities. Second, as previous studies show an association between social media use and depression [55], we measured historical activity levels of users (ie, active days) to capture effects that previous engagement may relate to both users’ emotions and their future engagement. We also measured users’ activity frequencies (eg, posting frequency) to capture their patterns of Twitter usage. Third, the covariates on observational bias were used to control for effects caused by incomplete observations. For example, a limited number of tweets were retrieved and used to measure emotions for a user. All variables on social capital, activity level, and observational bias were measured from users’ profile information and tweets collected in our first observation. Finally, as discussed above, we included the proportion and average durations of followees that were active in our second observation to capture the channel that followees’ emotions affect a user’s dropout through their effects on followees’ own dropouts.

### Model Estimations

#### Instrumental Variables Estimation in Linear Regression Model

We use standard 2-stage least squares estimators for linear probability models. In the first stage, we ran an auxiliary regression and predicted the endogenous variables (ie, an individual’s emotional state and network centrality) based on IVs and exogenous covariates. In the second stage regression, we substituted the endogenous variables of interest with their predicted values from the first stage. Estimation was conducted through the package of applied econometrics with R [71], and robust standard errors were computed.

#### Instrumental Variables Estimation in Survival Model

We used a Kaplan-Meier estimator [72] to estimate the survival function from data. Aalen’s additive hazards model [73] was used to estimate the effects of users’ attributes on the time to dropout. Compared with the proportional hazards models in which the ratios of hazard functions (ie, hazard ratios) for different strata were assumed to be constant over time [74], the additive model was more flexible and applied under less restrictive assumptions. To compute an IV estimator in an additive hazards model, we used a control function based approach which was proposed by Tchetgen et al [61]. The timereg package [75] was used for the implementation of the estimation algorithm. Standard errors were obtained through nonparametric bootstrap.

**Table 1:** Covariates used in estimations.

Control effect and covariate		Description
**Social capital**		
	#Followees	Number of total followees
	#Posts	Number of total posts, including tweets and retweets
	#Followers	Number of total followers
**Activity level**		
	Active days	Number of days from account creation to last posting
	#Followee/day	Average number of followees per day
	#Posts/day	Average number of posts per day
	#Followers/day	Average number of followers per day
**Observational bias**		
	#Tweets in use	Number of tweets in use to measure emotions
	#Followees in use	Number of followees whose attributes are used as instruments
**Alternative causal channel**		
	%Active followees	Proportion of followees being active between 2 observations
	Avg. duration of followees	Average days of followees being active between 2 observations

## Results

### Descriptive Statistics

**Figure 1:**
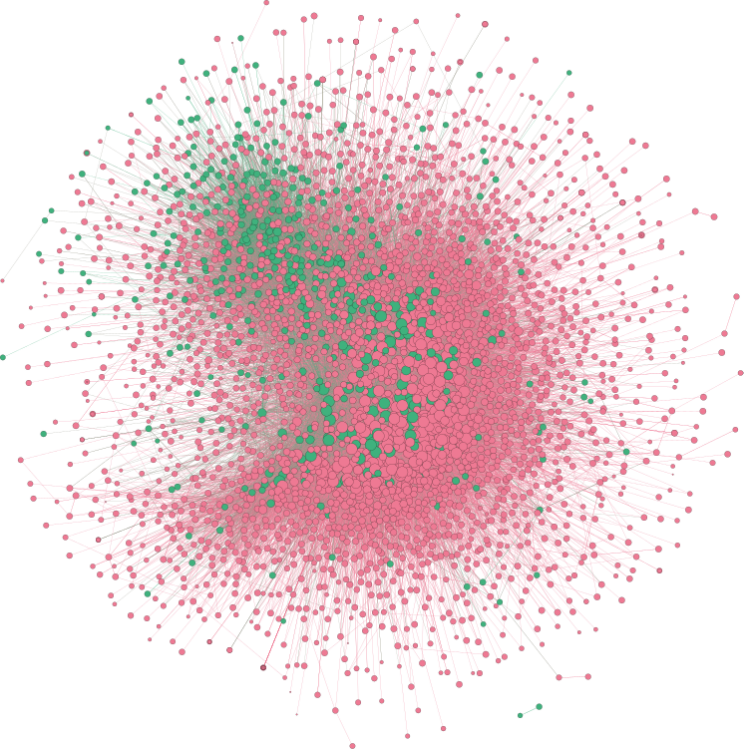
The who-follows-whom network among eating disorder users on Twitter. Node colors represent dropout statues, where the red color denotes dropout and the green color denotes nondropout. Node size is proportional to the in-coreness centrality.

We obtained 2906 users who posted more than 10 tweets (excluding retweets) and 50 words in our data, where 84.61% (*n*=2459) of users had no posting activities during our 2 observation periods. Among the 357 users who self-reported gender information in their Twitter profile descriptions, 84.0% of them (300) were female. The mean age was 17.3 years among ED users who self-reported age (*n*=1015). On the basis of the timestamps of account creation and last posting, we used the Kaplan-Meier estimator to estimate the “lifetime” of a user on Twitter, namely the duration from account creation to the last posting. The estimated median lifetime of these users on Twitter was 6 months. That is, one half of the entire cohort drops out at 6 months after creating an account. Figure 1 visualizes the social network between dropouts and nondropouts among ED users, laid out by the Fruchterman-Reingold algorithm [76]. We noted that users with the same dropout states tended to cluster together. Computing Newman’s homophily coefficient *r* [77] of this network by users’ dropout states, we found *r*=0.09 (*z*=16.84 and *P*<.001 compared with a null model, see Multimedia Appendix 1), suggesting that users with the same dropout states tended to befriend one another. See Multimedia Appendix 1 for details of data statistics.

### Estimation Results of Linear Probability Models

Table 2 shows estimated results in the linear models with 2 different IV specifications. In the first specification, we used all followees of a user to create IVs for the user’s attributes. The results are given in columns 2 to 3, in which both OLS and IV estimators show that positive emotions are associated with a higher probability of dropout (beta=.044; *P*=.007 and beta=.29; *P*<.001, respectively), with largely comparable coefficients for covariates. Compared with the OLS estimator, the IV estimator of the effect of emotions on dropout was remarkably stronger. The Wu-Hausman test further showed a significant difference between the OLS and IV estimators (*P*<.01), suggesting the presence of endogeneity. These results indicated that ignoring endogeneity in the OLS estimation leads to an underestimation of the effect of interest. Moreover, the *F* statistics in the first stage regressions showed that the relevance of IVs exceeds the conventional standard of *F*=10 [78], indicating the validity of our IVs.

**Table 2:** Estimated effects of emotions on dropout using ordinary least squares (OLS) and instrumental variables (IVs) models.

Variable	All followees (n=2906^a^)				Single way followees (n=2898^a^)			
	Ordinary least squares		Instrumental variables		Ordinary least squares		Instrumental variables	
	Beta	*P*^b^ value	Beta	*P*^b^ value	Beta	*P*^b^ value	Beta	*P*^b^ value
Emotions^c,d^	.044	.005	0.29	<.001	.064	<.001	.304	<.001
#Followees	−.0004	.01	−.0002	.18	−.0001	.11	−.0001	.22
#Posts	−.00000	.18	−0.00000	.35	−.00001	.06	−.00001	.14
#Followers	.00001	.53	.00001	.58	.00001	.7	0	.82
Active days	−.0003	<.001	−.0003	<.001	−.0003	<.001	−.0003	<.001
#Followee/day	.001	.01	.002	<.001	.001	.03	.002	.003
#Posts/day	.0002	.72	−.001	.38	.0001	.85	−.001	.32
#Followers/day	−.003	.01	−.005	<.001	−.003	.02	−.004	.002
#Tweets in use	−.00004	.002	−.00004	.003	−.00003	.03	−.00003	.03
#Followees in use	.0004	.03	.0002	.38	0	.96	−.0001	.41
%Active followees	−1.159	<.001	−.812	<.001	−.939	<.001	−.655	<.001
Avg. duration of followees	.001	.004	.001	.16	.001	.005	.0005	.19
Constant	1.27	<.001	1.273	<.001	1.246	<.001	1.251	<.001

^a^The numbers of samples that are used in estimations.

^b^*P* values are computed based on heteroscedasticity-consistent standard errors.

^c^*F* statistic tests the significance of the instrument from a first-stage regression of a user’s emotions on followees’ emotions (ie, the instrument) and the rest of the covariates, where *F=440.26 (P<.001)* when all followees are used to build an instrument and *F=158.21 (P<.001)* when only single way followees are used.

^d^Wu-Hausman (H) statistic tests the difference in estimates between OLS and IV, where *H=42.24 (P<.001)* when all followees are used to build an instrument and *H=14.54 (P<.001)* when only single way followees are used. Rejecting the null hypothesis suggests the presence of endogeneity.

Columns 4 to 5 show results of the second IV specification in which only single way followees are used to create IVs. Users who have no single way followees are excluded as instruments for these users’ attributes were not available. Thus, the number of observations decreased as compared with that in the first IV specification. Moreover, as data on a smaller number of friends were used in the second IV specification, the relevance of IV became weaker but still passed the conventional test in the first stage regression. Despite such changes, the 2 specifications produced largely similar results. Computing Wald tests of equality of coefficients between the 2 IV models, we found that the estimated effects of emotions on dropout were statistically the same across different IV specifications (*P*=.8), potentially suggesting robustness of the results.

Note that network centrality was excluded from the linear models. This is because many users had dropped out long before our first observation (see Figure S2 in Multimedia Appendix 1), and the social networks of such users might largely change from the dates of their dropouts to our first observation. For example, a user might be followed by new followers when these followers were unaware of the dropout of this user. In these cases, network centralities in the future are used to explain dropouts in the past, which can produce misleading results in the linear models. In fact, including network centrality in the above linear models produces statistically insignificant effect of centrality on the dropout decision, confirming our argument that network centrality is irrelevant to the binary decision to drop out or not.

### Estimation Results of Survival Models

In the survival models, we only considered users who were active past our first observation period, so as to examine the effect of network centralities in our first observation on users’ activities in the second observation period. Table 3 shows mean coefficients of emotions and network centrality in the survival models. All models are estimated controlling for the full list of covariates but are omitted from the tables because of space concerns. The complete results are available from the authors. Following [61], the effects of all covariates are assumed to be time dependent in estimations. Both the standard and IV models on the identical interval censored data show that (a) positive emotions lead to a shorter survival time (*P*<.05 in the IV model) and (b) a core position in social networks is associated with a longer survival time (*P*<.05 in both models). Estimations on the personalized interval censored data and using different IV specifications gave similar results. The strong relevance of IVs in the first stage regressions confirms the validity of IVs across different models. A comparison of results between the linear and survival models further shows that these models have consistent estimators for the effect of emotions on dropout, namely positive emotions increase the likelihood to drop out.

**Table 3.** Estimated effects of emotions and centrality on survival time using Aalen’s additive hazards models.

Variable^a,b^		All followees (n=447^c^)		Single way followees (n=445^c^)	
		Standard (95% CI^d^)	IV^e^ (95% CI)	Standard (95% CI)	IV (95% CI)
**Identical interval censoring**					
	Emotions	−0.018 (−0.037 to 0.0002)	−0.043 (−0.083 to −0.004)	−0.018 (−0.036 to 0.0006)	−0.061 (−0.116 to −0.011)
	Centrality	0.001 (0.0008 to 0.0011)	0.001 (0.0007 to 0.0011)	0.001 (0.0008 to 0.0011)	0.001 (0.0006 to 0.0011)
**Personalized interval censoring**					
	Emotions	−0.016 (−0.034 to 0.0031)	−0.038 (−0.08 to 0.002)	−0.015(−0.034 to 0.0026)	−0.056 (−0.115 to −0.007)
	Centrality	0.001 (0.0008 to 0.0011)	0.001 (0.0008 to 0.0012)	0.001 (0.0008 to 0.0011)	0.001 (0.0007 to 0.0011)

^a^*F*-statistic tests the joint significance of the 2 instruments from a first-stage regression of a user’s emotions on followees’ emotions and followees’ centralities (ie, the instruments) plus the rest of the covariates, where *F=66.11 (P<.001)* when all followees are used to build instruments and *F=34.99 (P<.001)* when only single way followees are used.

^b^*F*-statistic tests the joint significance of the 2 excluded instruments from a first-stage regression of a user’s centrality on followees’ emotions and followees’centralities (ie, the instruments) plus the rest of the covariates, where *F=27.85 (P<.001)* when all followees are used to build instruments and *F=12.62 (P<.001)* when only single way followees are used.

^c^The numbers of samples that are used in estimations.

^d^CIs for coefficients are obtained from 1000 bootstrap replicates. A coefficient is significant at *P<.05* if 0 is not in 95% CIs.

^e^IVs stands for instrumental variables.

### Underlying Connection Between Emotions and Dropout

To better understand the relationships between emotions and dropout, we examined posting interests among users with different dropout statuses and emotional states based on hashtags used in users’ tweets (see Multimedia Appendix 1 for details). We found nondropouts were interested in advocating a thin ideal (eg, using hashtags “mythinspo” and “skinny4xmas”) and promoting a pro-ED identity (eg, “edlogic” and “beautiful”). In contrast, dropouts engage in discussing their health problems (eg, “selfharmprobz,” “bulimicprobz,” and “anorexicprobz”) and offering emotional support for others (eg, “anasisters” and “stayingstrong”), which implies a tendency of these users to recover from disorders [20-22]. Similarly, we split all ED users into 3 equal-size sets based on their emotional scores and examined hashtags used by each set of users. We found that users with negative emotions often engage in promoting thin ideals (eg, “bonespo” and “mythinspo”), showing largely overlapping interests with the nondropouts. In contrast, users with neutral and positive emotions were more interested in discussing their health problems (eg, “anorexicprobz” and “bulimicprobz”), opposing pro-ED promotions (eg, “reversethinspo”), and encouraging healthier body image and behaviors (eg, “fitfam” and “fitness”), showing similar interests with the dropouts. See Multimedia Appendix 1 for more detailed lists of hashtags.

Measuring the Spearman rank correlation *ρ* between pairwise lists of hashtags posted by users with a given state (eg, dropped out or not, and positive or negative), we found a positive correlation between negative users and nondropouts in hashtag usage (*ρ*=0 *.* 36; *P*=.003 in Table 4), indicating similar posting interests among these users. A similar pattern occurred between positive users and dropouts. In contrast, users with other pairs of states showed a negative correlation or noncorrelation in hashtag usage, indicating their discrepancies in posting interests. Note that all tags in 2 lists *Di* and *Dj* are considered in computing the correlation *ρ* (*Di*, *Dj*); tags in each list are ranked by TF-IDF scores [79] and the TF-IDF score of tag *t* in list *Di* is 0 if *Di* does not contain *t*. These results revealed a possible underlying connection between positive emotions and dropout. Compared with users with positive emotions, those with negative emotions had more similar interests to active members (ie, nondropouts) in the ED community. Finding similarities with other members in a community can enhance a sense of belonging to the community and positively increase intention to engage in community activities [33,37]. Therefore, it is not surprising that negative users are less likely to drop out than positive users in our estimations.

**Table 4:** Spearman rank correlations between pairwise lists of hashtags posted by users with a given dropout state and by users with a given emotional state, respectively.

Group	Negative (n=61^a^)	Neutral (n=108)	Positive (n=110)
Nondropout (n=54^a^)	0.36 (*P*=.003)^b^	−0.21 (*P*=.03)	−0.66 (*P*<.001)
Dropout (n=227)	−0.33 (*P*<.001)	−0.04 (*P*=.57)	0.12 (*P*=.07)

^a^The number of hashtags posted by users with a given state.

^b^The Spearman correlations *ρ* of hashtags posted by users with different dropout and emotional states, where *ρ* ranges from *−1* to *1* and 0 indicates no correlation. *P* values testing for noncorrelation are reported in parentheses.

## Discussion

### Principal Findings

This study provided the first estimates of the effects of personal emotions and interpersonal social networks on dropout in online ED communities. This study has several strengths. First, we based our analysis on the incentive theory to explore determinants of users’ online behaviors (ie, dropout), allowing us to study users’ behaviors in a more systematic way than most previous studies that often focus on a single type of determinant (eg, individual attributes [35,36] or social attributes [42,43,67]). Second, we used automated sentiment analysis techniques to measure users’ emotions and network analysis methods to quantify users’ sociometric statuses in an online community, leading to higher efficiency than traditional research methods such as surveys [35,37,39,41,48]. Finally, we applied an IV approach to both linear probability and survival models, which enabled us to achieve a more consistent estimate of human behavior in online settings than traditional methods (eg, OLS) used in previous studies [39,41,47]. Overall, we found that positive emotions increased the likelihood of dropout in ED individuals and accelerated the dropout process on Twitter. In contrast, a central position in the social network of ED individuals at an earlier stage was associated with prolonged participation of an individual at a later stage. These findings were verified across a variety of robustness checks.

Despite differences in methodology, our findings aligned with previous studies in psychological and social media research [5,33,35]. Our results suggested that ED users with negative emotions had high levels of participation on Twitter. This aligned with previous survey studies on social media use (eg, Facebook use), where people with social anxiety and shyness (ie, personality traits that are often correlated with multiple negative emotions such as feeling lonely, isolated, and unhappy [80]) were found to spend more time online [35,48,81]. An explanation for this is the online disinhibition effect [82]. Specifically, because of anonymity in online interactions, people with social inhibitions (eg, those who are socially anxious or shy and those with a stigmatized health problem [83]) might be more willing to share personal feelings and reveal themselves in online interactions than offline interactions to meet their social and intimacy needs [48]. Additional analyses on users’ posting interests revealed that users with negative emotions share similar interests with active users. This allowed us to confirm the validity of our results via the social capital theory [39,40], namely sharing common attributes (eg, interests and vision) with other members can enhance a sense of belonging and positive feeling toward a community, which drives people to actively engage in the community.

Consistent with positive associations between network centrality and active participation in other online communities [67,70], we find that central users in the social network of an ED community tend to have a longer-lasting participation in the community. This result is expected for several reasons. First, users who are centrally embedded in a group have a relatively high number of social ties with other members, which can lead these users to feel being socially accepted and approved, as well as a strong sense of belonging to the group. Previous studies have consistently shown that recognition from other members and identification within an online community increase an individual’s commitment to the community [34,39-41]. Finally, information shared by central users is likely to spread to the majority of a community through social ties, and their central positions in the community may promote other members to trust such information [70]. This implies that central users have a greater potential than peripheral users in influencing members’ opinions, emotions, and behaviors in online communities [84]. Thus, compared with peripheral users, feeling influential may provide an additional incentive for central users to continue participating.

In line with previous studies on online ED communities [15,17,21], we found that ED users on Twitter have different stances on EDs, where users with negative emotions often share pro-ED content and those with positive emotions often share prorecovery content. As pro-ED content often contains thin-ideal images and harmful tips for weight loss and control [8,24,25], this result aligned with clinical evidence on ED treatment showing that more emotional distress is associated with a higher risk to learn and develop dysfunctional coping behaviors among ED sufferers [5]. Thus, as suggested by previous studies [85], engaging in pro-ED content may serve as a coping mechanism to deal with emotional pressures and stress of EDs. A possible explanation for the association between engaging in harmful online content and coping with stress is sensation seeking [86], a basic personality trait defined as the seeking of varied, novel, complex and intense sensations and experiences, and the willingness to take risks. Several studies have shown that sensation seeking is prominent in adolescence (ie, the age at which disordered eating often develops [1]) and closely related to pathological internet use, such as use of violent sites [87] and internet dependence [88].

Our study also offered new insights into online ED communities. First, ED users have a high dropout rate (85% in our sample) and a short lifespan between an account creation to lost posting on Twitter (with 6 months of median time to drop out). This aligns with views of online ED communities as hidden, secretive groups [30], but also indicates the dynamic characteristics of these communities. Second, users who discuss their health problems and share prorecovery content (ie, prorecovery users) have lower levels of posting activities (ie, a higher dropout rate) than those who share pro-ED content (ie, pro-ED users) on Twitter. This can be explained as follows. Owing to common interests in EDs, prorecovery and pro-ED groups are likely to be connected in the same social networks, and content shared within a group is hence likely to be visible to the other group. However, exposure to content from the antagonist group can have distinct effects in pro-ED and prorecovery groups. Exposure to pro-ED content is harmful for prorecovery users and can impede their recovery process [3,24], whereas exposure to prorecovery content can instead stimulate harmful behaviors in pro-ED users (eg, actively sharing pro-ED content) [21]. Thus, prorecovery users might tend to leave such an online community to avoid a risk of further deterioration or relapse. Our finding may also explain why pro-ED content is found being more pervasive than prorecovery content across social media sites [15,17,21], for example, almost 5 times in terms of unique publishers on Tumblr [15]. Finally, ED users tend to connect with others with the same dropout states on Twitter. This implies that whether an individual drops out from online communities depends on whether others in the individual’s social networks drop out. In other words, dropout in online ED communities is not only a function of individual experience or individual choice but also a property of group interactions, such as homophily [89] and social contagion effects [59].

### Implications

Our findings are of practical relevance to the promotion of public health over social media. First, the decision to maintain active participation in an online community can be caused by intrinsic and extrinsic characteristics or traits of the participants, such as personal emotions, interests, and social networks. Such self-selection bias can lead to the sample not being representative of the whole population, and hence researchers need to consider both active and dropped-out users for a well-rounded picture of online health communities. This is particularly important for public health officials to make special efforts to reach these dropouts and offer more intensive support when they are trying to recover. Second, high attrition rates are often regarded as negative outcomes in online interventions, particularly in those delivered over a purposely designed website [11,31,32]. However, this may or may not be the case in interventions over general social media sites (eg, Twitter) depending on how targeted populations use these sites. For example, when an intervention is delivered in an online community in which members often shared harmful content, a high attrition rate (ie, members dropping out of the harmful community) may not be a negative outcome. Using automated data-mining techniques to track users’ behaviors (eg, emotions and posting interests), as used in this study, can provide more detailed information about people’s use of online health communities and improve our understanding of attrition in online interventions. Third, interventions that recommend content containing positive emotions to ED users (not limited to ED-related content but more general content containing happiness and inspiration) may reduce their engagement in a harmful online community. This aligns with Fredrickson’s broaden-and-build model which argues that cultivating positive emotions is useful to prevent and treat mental health problems [90]. Finally, intervention strategies could be tailored for different individuals depending on their positions in the social network of an online community. For example, identifying central individuals as change agents might enhance the efficacy and cost-effectiveness of an intervention because of their greater influence potential through larger numbers of social ties [91] and also their longer-lasting effects through longer-term participation in the community.

### Limitations

First, we recognized that self-diagnosis information on Twitter might be itself self-censored by users to align with their personality traits and perceptions of their audience on the platform. People may not use tags such as “eating disorder” to self-report their experience of illness and would be excluded by our collection methods. Also, although over 208,000 users and over 241,000,000 tweets are studied in this study, a small sample of rich social media data is used to explore the attrition of ED communities on Twitter. Thus, generalization of our results to all ED-related online communities should be cautious. Second, our measures of dropout are based on posting activity, whereas some people primarily use Twitter to receive outside information but rarely post their own information. We have little activity data on these users and hence less understanding of the characteristics of their dropout. This thus raises important issues that need further research to enhance our understanding of attrition in online health communities, such as consensus and clarity about the definition of dropout. Third, our study focused on the Twitter platform, without validation on other platforms. However, stopping using a platform can be related to the attractiveness of the platform. Hence, future research is also in need to examine many other factors that we did not explore but can affect dropout on social media, such as individual personality, physical health states, perceptions, and purposes of using a particular social media platform. Fourth, user accounts on a social media site are often not unique—an individual may have multiple accounts on the same site. Thus, we cannot be certain whether individuals who stopped using an account will engage in the same or similar online communities through other accounts. In other words, the dropout of a user account may not necessarily imply that an individual abandons a specific identity (eg, pro-ED) shared within a community. Finally, although our methodology allows us to establish a causal link from emotions to dropout behavior, it offers limited insights into the pathways through which this link exactly operates, and future work is needed to explore such issues in detail.

### Conclusions

This study presented a systematic characterization of attrition in an ED community on Twitter. Our analysis offered the first attempt toward the estimation of the effects of personal emotions and network centrality on dropout behaviors in individuals affected by ED on Twitter. Our results provided new insights into the trajectories that ED communities develop online which can help public health officials to better understand individual needs in using online ED communities and provided tailored support for individuals with different needs.

## Appendix

Multimedia Appendix 1 Supplementary information for estimating determinants of attrition in eating disorder communities on Twitter: an instrumental variables approach.

## Abbreviations

API: application programming interface
ED: eating disorder
IV: instrumental variable
OLS: ordinary least squares
