# Supplementary material for: Estimating Determinants of Attrition in Eating Disorder Communities on Twitter: An Instrumental Variables Approach
Source: J Med Internet Res. 2019 May 3;21(5):e10942. doi: 10.2196/10942 (PMC6533043; doi:10.2196/10942)
Supplement: Multimedia Appendix 1 [file jmir_v21i5e10942_app1.pdf]

Supplementary Information for
Estimating Determinants of Attrition in Eating Disorder Communities on Twitter: An Instrumental Variables Approach

Tao Wang, Emmanouil Mentzakis, Markus Brede, and Antonella Ianni

Contents

1 Data Statistics **1**

2 Null Model **3**

3 Specifications of Data Censoring Methods **4**

4 Posting Interests of Users **4**

List of Figures

S1 Diagram of data collection and analysis procedures. 2
S2 Number of users along with time points when users created a Twitter account and posted the last tweet. 3
S3 Demographics of ED users, extracted from users' self-reports in their Twitter profile descriptions. A dotted line marks the mean of a corresponding statistic and the sample size for a statistic is reported in parentheses. 4
S4 Kaplan-Meier estimations of survival time. 5
S5 The co-occurrence network of the most popular hashtags used by all ED users. Each node is a hashtag, and node size is proportional to the number of users who posted the tag. Node color is assigned based on the frequency of a tag so that high frequency is darker and low frequency is lighter. Edge width is proportional to the number of co-occurrences of two attached tags in tweets. 5

List of Tables

S1 Descriptive statistics of users by dropout and non-dropout states. 2
S2 The most popular hashtags used by ED users, grouped by users' dropout states. . . 6
S3 The most popular hashtags used by ED users, grouped by users' emotional states. . 6

1 Data Statistics

Figure S1 shows a diagram of our data collection and analysis processes. Table S1 shows descriptive statistics of users stratified by dropout states that are observed in our second observation period. The differences between dropouts and non-dropouts are measured with the Mann-Whitney U-test. The U-test is a nonparametric test with the null hypothesis that the distributions of two

Table S1: Descriptive statistics of users by dropout and non-dropout states.

Attributes	All ($n = 2,906$)		Non-dropout ($n = 447$)		Dropout ($n = 2,459$)		U-test	
	Mean	SD	Mean	SD	Mean	SD	z^b	P^c
Emotions	-0.09	0.36	-0.13	0.33	-0.08	0.37	-3.06	.03
Centrality	29.35	16.22	35.89	15.25	28.16	16.11	9.26	< .001
#Followees	309.34	483.29	533.19	862.29	268.64	360.69	10.32	< .001
#Posts	899.62	2225.44	2298.01	4566.24	645.42	1281.62	16.02	< .001
#Followers	308.15	752.74	656.25	1422.54	244.87	525.09	13.64	< .001
Active days	348.20	403.06	732.17	524.86	278.40	332.03	18.43	< .001
#Followee/day	4.16	19.07	1.75	4.87	4.60	20.60	-8.69	< .001
#Posts/day	4.41	7.54	3.85	5.82	4.51	7.81	-1.13	1
#Followers/day	2.66	8.23	1.57	3.03	2.85	8.84	-4.97	< .001
#Tweets in use	614.43	827.33	1244.17	1078.79	499.96	715.65	16.47	< .001
#Followees in use	242.13	366.69	405.27	639.19	212.47	280.92	9.56	< .001
%Active followees	0.43	0.16	0.53	0.17	0.41	0.15	13.70	< .001
⟨Followees' durations⟩[a]	193.18	83.24	243.42	89.64	184.05	78.65	13.23	< .001
⟨Followees' emotions⟩[a]	0.06	0.16	0.01	0.16	0.07	0.16	-8.75	< .001
⟨Followees' centralities⟩[a]	29.38	9.04	28.04	8.70	29.62	9.08	-3.65	.004

[a]⟨Followee x⟩ denotes the average values of a user's followees in terms of statistics x.
[b]A z-score measures the extent of a variable in non-dropout group being larger than that in dropout group.
[c]P-values for two-tailed tests with Bonferroni correction.

populations are equal. This test does not need the assumption of a specific distribution in data (e.g., a normal distribution in the t-test), well suitable for statistics on social media that often follow a non-normal (e.g., power law) distribution [1]. For intuitive comparisons, we report a standardized U as a z-score. Moreover, the Bonferroni correction is used to counteract the problem of multiple comparisons. Compared to dropouts, non-dropouts show more negative emotions and higher network centralities. The network centralities are measured based on a following network containing 208,063 nodes and 1,347,056 directed edges. All nodes are connected in a single weakly connected component and the average degree of the network is 6.5.

Figure S2 shows details on dates when users joined and dropped out on Twitter. Most users were active during 2012 to 2014, during which 1,944 users (67%) joined Twitter. Two notable peaks in the curve of last posting time occur at the dates of our two observations. The first peak indicates that some users were lost to follow up (e.g., accounts were deleted), and the second peak indicates

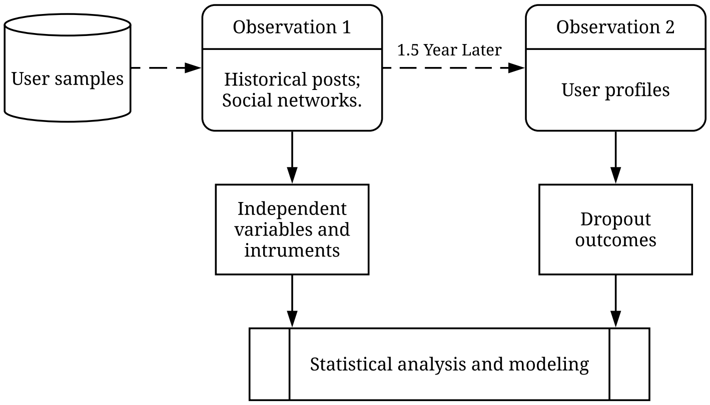

Figure S1: Diagram of data collection and analysis procedures.

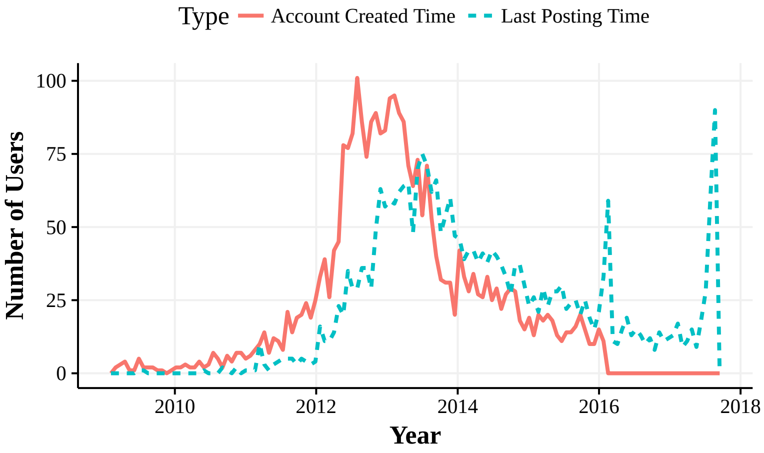

Figure S2: Number of users along with time points when users created a Twitter account and posted the last tweet.

that many users were still actively posting tweets until our observations ended.

Figure S3 shows demographic information of ED users, extracted from users' Twitter profile descriptions using regular expressions. To avoid noise (i.e., extremely small or large values), we only consider users whose values are in the 95% confidence intervals of the whole distribution of a statistic (except for gender). We obtain 357 users who self-reported gender information and 84% of these users ($n = 300$) are female (see Figure S3(a)). There are 1,030 users who reported their ages in total. After excluding those with extremely small and large values of age, Figure S3(b) shows the distribution of age among ED users ($n = 1,015$), and the mean age of these users is 17.3. The majority of females and young ages in the ED users align with clinical evidence that ED are often developed among young females [2, 3]. Figures S3(c) and (d) further show the distributions of height (with the mean of $\mu = 165.1$cm) and weights ($\mu = 57.6$kg for current weight and $\mu = 49.4$kg for goal weight) among ED users. Comparing the distributions of weights, we see that the values of goal weights are smaller than those of current weights. This implies that most ED users attempt to lose weight. Moreover, we calculate BMI (Body Mass Index) for users who reported information about both height and weight. Figure S3(e) shows the distributions of users' current ($\mu = 21.1$) and goal ($\mu = 18.4$) BMIs. Compared to the reference values of BMI for girls at the age of 17.3 years from World Health Organization (WHO) [4], we find that 58% of users ($n = 574$ among 991 users) have a current BMI lower than 21.1 (the reference value of median BMI), and 55% of users ($n = 619$ among 1,123 users) have a goad BMI lower than 18.5 (the reference BMI for underweight). Note that we do not include users' demographic attributes in our estimation models due to a low fraction of users who have these attributes (e.g., only 357 of 3,380 users with gender information).

2 Null Model

We use a null model [5] to test the statistical significance of the homophily pattern. Specifically, we randomly shuffle users' dropout states and re-measure homophily coefficients r [6] based on the shuffled states. These coefficients can be viewed as observed values of a random variable. Repeating this procedure 3,000 times, we yield the empirical distribution of homophily coefficients with a mean of $\mu = 0$ and a standard deviation of $\sigma = 0.005$. The z-score for the observed homophily (i.e., $r = 0.09$ in the main text) under this baseline distribution is $z = 16.84$ and

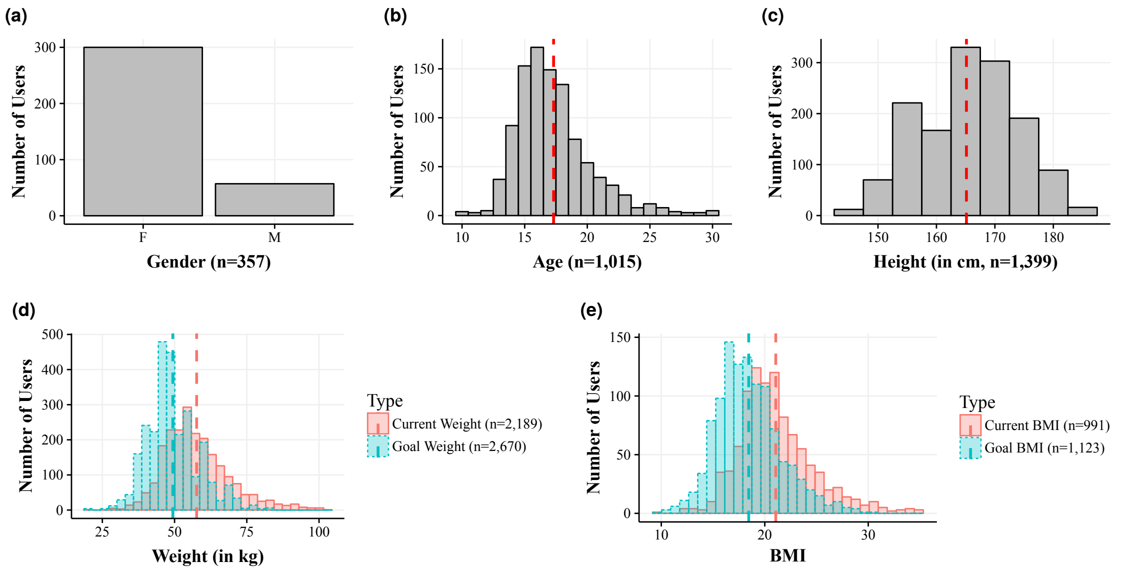

Figure S3: Demographics of ED users, extracted from users' self-reports in their Twitter profile descriptions. A dotted line marks the mean of a corresponding statistic and the sample size for a statistic is reported in parentheses.

$P < .001$, suggesting the presence of homophily.

3 Specifications of Data Censoring Methods

We tune the parameters of our data censoring methods based on users' activities before and after our first observation. We apply each censoring method with different parameters to data on users' activities before our first observation to estimate users' dropout states, and choose parameters that achieve the best agreement between the estimated dropout states and the observed states in our second observation. By setting $\pi \in [1, 300]$ days in the identical-interval censoring method, we find the optimal parameter being $\pi = 101$, with Cohen's $\kappa = .68$ of the estimated dropout states and the observed dropout states of users. Such good agreement illustrates the effectiveness of the censoring approach. Similarly, by searching in a parameter space of $\pi \in [0, 200]$ days and $\lambda \in [0, 1]$, we find the optimal parameters in the personalized-interval censoring method being $\pi = 161$ and $\lambda = 0.6$, with Cohen's $\kappa = .68$ as well. We use these parameters censor the dropout states of users who were active in the second observation. Figure S4 shows the Kaplan-Meier curves of users' survival time from our first observation until our second observation using the two censoring methods. The median survival time of users is 13 months in both methods, and no significant difference is found between the two types of censorships ($P = .93$ in a log-rank test).

4 Posting Interests of Users

To better understand the relationship between emotions and dropout, we examine associations of interests among users with different dropout statuses and emotional states. This follows past evidence that community interest is the primary motivating factor for participation in online communities [7] and people's concerns/interests reflect their emotional states [8]. Since hashtags are explicit topic signals on Twitter and have been shown to strongly indicate users' interests [9], we characterize users' interests based on hashtags used in their tweets.

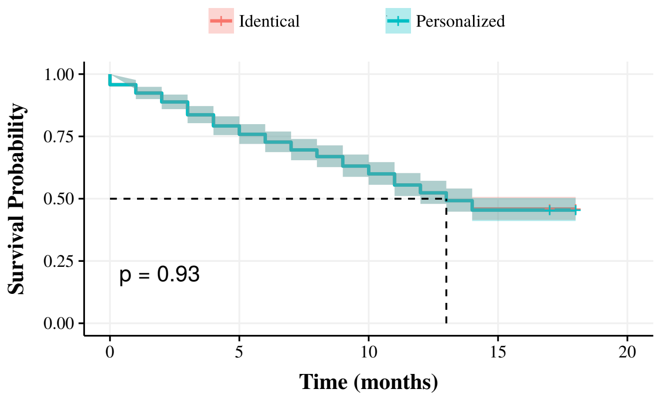

Figure S4: Kaplan-Meier estimations of survival time.

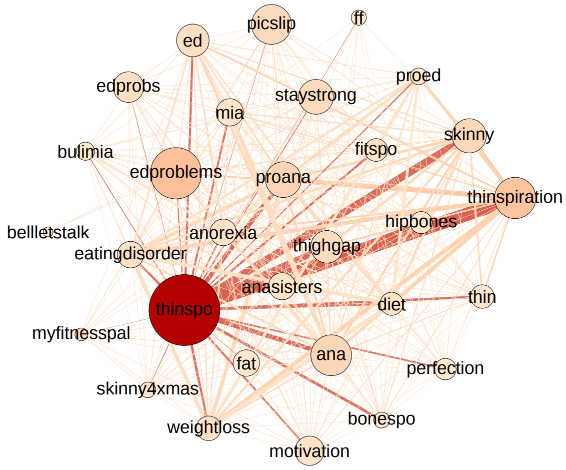

Figure S5: The co-occurrence network of the most popular hashtags used by all ED users. Each node is a hashtag, and node size is proportional to the number of users who posted the tag. Node color is assigned based on the frequency of a tag so that high frequency is darker and low frequency is lighter. Edge width is proportional to the number of co-occurrences of two attached tags in tweets.

We first examine the prevalent topics of interest for the entire ED community. To capture relationships between different topics, we build an undirected, weighted hashtag network based on the co-occurrences of hashtags in tweets posted by ED users, where an edge is weighted by the co-occurrence count of two attached tags. To filter out noise from accidental co-occurrences and spam, we only consider hashtags used by more than 50 distinct users and observed in more than 50 tweets, resulting in a network of 312 nodes and 7,906 edges. Figure S5 shows the co-occurrence network of the most popular hashtags of interest for ED users. We observe that topics on promoting a thin ideal (e.g., “thinspo” and “thinspiration”) are very prevalent in the community.

We then examine interests of users with different dropout states. We split ED users into two sets based on their dropout states in our second observation, and extract hashtags from tweets posted by each set of users. Again, tags that are used by less than 50 users and occur in less than

Table S2: The most popular hashtags used by ED users, grouped by users' dropout states.

Dropout states	Hashtags[a]
Non-dropout	legspo, mythinspo, skinny4xmas, bonespo, goals, edlogic, eatingdisorders, edthoughts, ribs, bones, depressed, depression, edprobs, collarbones, bulimia, promia, replytweet, beautiful, anorexia, thin, hipbones, legs, ednos, ed, thighgap, weightloss, skinny, proed, selfharm, perfection, mia, thinspiration, perfect, proana, diet, eatingdisorder
Dropout	goaway, stopbullying, worthless, selfharmprobz, ew, anasisters, yay, oneday, reasonstobefit, bulimicprobz, anorexicprobz, fact, disgusting, thankgod, willpower, tweetwhatyoueat, wow, toofat, jealous, thankyou, true, anasister, anafamily, starveon, gross, teamfollowback, fuck, icandothis, tired, edfamily, relapse, stayingstrong

[a]Tags for each state are ranked in a decreasing order based on the TF-IDF score of a tag, which is calculated by the ratio of the number of users who post the tag and have a given dropout state to the number of users who post the tag in the whole user sample (i.e., regardless of users' dropout states).

Table S3: The most popular hashtags used by ED users, grouped by users' emotional states.

Emotional states	Hashtags[a]
Negative	bonespo, mythinspo, edlogic, bulimia, depression, starve, eatingdisorders, anxiety, anorexia, skinny4xmas, depressed, edprobs, proed, ribs, bones, edproblems, thinspo, edthoughts, thinspiration, selfharm, fuck, goals, thin, edgirlprobs, fat, sad, skinny, ednos, realityproject, ana, eatingdisorder, fatass, hipbones
Neutral	awkward, anorexicprobz, fast, mylife, bulimicprobz, please, sorry, fuckyou, myfitnesspal, ew, skinny4xmas, legspo, edfamily, gross, anafamily, ugh, ednos, workout, goals, replytweet, tmi, fatass, reversethinspo, edprobz, anaproblems, failure, flatstomach, fact, binge, fatty, fasting, suicide, depressed
Positive	eatclean, fitfam, inspiration, reasonstobefit, ff, noexcuses, fitness, loveit, winning, anasister, tweetwhatyoueat, twye, keepgoing, success, jealous, want, fitspo, beforeandafter, retweet, excited, proud, reasonstoloseweight, abcdiet, fail, justsaying, rt, motivated, workout, stayingstrong, love, myfitnesspal

[a]Tags for each state are ranked in a decreasing order based on the TF-IDF score of a tag, which is calculated by the ratio of the number of users who post the tag and have a given emotional state to the number of users who post the tag in the whole user sample (i.e., regardless of users' emotional states).

50 tweets in each set are excluded. To adjust tags that are popular in general, we use TF-IDF [10] to rank the specificity of a tag in each set of users. Table S2 lists the most representative hashtags in each user set, in which we find that users with different dropout states display distinct interests online. Non-dropouts are interested in advocating a thin ideal (e.g., "mythinspo" and "skinny4xmas") and reinforcing a pro-ED identity (e.g., "edlogic" and "beautiful"). In contrast, dropouts engage more in discussing their health problems (e.g., "selfharmprobz", "bulimicprobz", "anorexicprobz" and "relapse") and offering emotional support for others (e.g., "anasisters" and "stayingstrong"), which implies a tendency of these users to recover from disorders [11, 12, 13]. Together, these results imply that pro-recovery users are more likely to drop out than pro-ED users. A comparison of interests between each individual set and the entire community (see Figure S5) further shows that the non-dropouts have dominated the topics of discussions within the community. This is expected because the non-dropouts have prolonged participation, with 732.17 active days on average compared to 278.40 days of the dropouts (see Table S1).

Similarly, we split ED users into three equal-size sets based on their emotional scores and obtain the most representative hashtags among each set of users in Table S3. The results show that users with negative emotions more engage in promoting thin ideals (e.g., "bonespo" and "mythinspo"), showing largely overlapping interests with the non-dropouts. In contrast, users with neutral and positive emotions are more interested in discussing their health problems (e.g., "anorexicprobz" and "bulimicprobz"), opposing pro-ED promotions (e.g., "reversethinspo") and encouraging healthier body image and behaviors (e.g., "fitfam" and "fitness"), showing similar

interests with the dropouts.

To further quantify the similarity (or association) of posting interests between users with a dropout state and those with a emotional state (as identified in Tables S2 and S3), we measure the Spearman rank correlation r between pairwise lists of hashtags posted by users with a given state (e.g., dropped-out or not, and positive or negative). We use the Spearman correlation because (i) it is more robust to scaling of data than other measures (e.g., cosine similarity) and (ii) it does not assume that datasets follow a specific distribution (e.g., a normal distribution in the Pearson correlation). The results of correlations are shown in Table 4 of the main text.

References

[1] H. Kwak, C. Lee, H. Park, and S. Moon, "What is twitter, a social network or a news media?," in *Proceedings of the 19th international conference on World wide web*, pp. 591–600, ACM, 2010. [DOI: 10.1145/1772690.1772751].

[2] D. S. Abebe, L. Lien, and T. von Soest, "The development of bulimic symptoms from adolescence to young adulthood in females and males: A population-based longitudinal cohort study," *International Journal of Eating Disorders*, vol. 45, no. 6, pp. 737–745, 2012. [PMID: 22886952] [DOI: 10.1002/eat.20950].

[3] A. P. Association *et al.*, *Diagnostic and statistical manual of mental disorders (DSM-5®)*. American Psychiatric Pub, 2013. [DOI: 10.1176/appi.books.9780890425596.529303].

[4] WHO, "Bmi-for-age girls," *World Health Organization (WHO)*, 2018-10-23. URL:`http://www.who.int/growthref/bmifa_girls_5_19years_z.pdf?ua=1`. (Archived by WebCite at `http://www.webcitation.org/73NvLTjZr`).

[5] M. E. Newman and M. Girvan, "Finding and evaluating community structure in networks," *Physical review E*, vol. 69, no. 2, p. 026113, 2004. [PMID: 14995526] [DOI: 10.1103/PhysRevE.69.026113].

[6] M. E. Newman, "Mixing patterns in networks," *Physical Review E*, vol. 67, no. 2, p. 026126, 2003. [DOI: 10.1103/physreve.67.026126].

[7] C. M. Ridings and D. Gefen, "Virtual community attraction: Why people hang out online," *Journal of Computer-Mediated Communication*, vol. 10, no. 1, pp. 00–00, 2004. [DOI: 10.1111/j.1083-6101.2004.tb00229.x].

[8] J. D. Mayer and G. Geher, "Emotional intelligence and the identification of emotion," *Intelligence*, vol. 22, no. 2, pp. 89–113, 1996. [DOI: 10.1016/s0160-2896(96)90011-2].

[9] L. Weng and F. Menczer, "Topicality and impact in social media: Diverse messages, focused messengers," *PloS one*, vol. 10, no. 2, p. e0118410, 2015. [PMID: 25710685] [DOI: 10.1371/journal.pone.0118410].

[10] K. Sparck Jones, "A statistical interpretation of term specificity and its application in retrieval," *Journal of documentation*, vol. 28, no. 1, pp. 11–21, 1972. [DOI: 10.1108/eb026526].

[11] M. Wolf, F. Theis, and H. Kordy, "Language use in eating disorder blogs: Psychological implications of social online activity," *Journal of Language and Social Psychology*, 2013. [DOI: 10.1177/0261927X12474278].

[12] E. Yom-Tov, L. Fernandez-Luque, I. Weber, and S. P. Crain, "Pro-anorexia and pro-recovery photo sharing: a tale of two warring tribes," *Journal of medical Internet research*, vol. 14, no. 6, p. e151, 2012. [PMID: 23134671] [DOI: 10.2196/jmir.2239].

[13] E. J. Lyons, M. R. Mehl, and J. W. Pennebaker, “Pro-anorexics and recovering anorexics differ in their linguistic internet self-presentation,” *Journal of psychosomatic research*, vol. 60, no. 3, pp. 253–256, 2006. [PMID: 16516656] [DOI:10.1016/j.jpsychores.2005.07.017].
